# Are we doing enough? Evaluation of the Polio Eradication Initiative in a district of Pakistan's Punjab province: a LQAS study

**DOI:** 10.1186/1471-2458-10-60

**Published:** 2010-02-09

**Authors:** Muhammad Umair Mushtaq, Muhammad Ashraf Majrooh, Mohsin Zia Sana Ullah, Javed Akram, Arif Mahmood Siddiqui, Mushtaq Ahmad Shad, Muhammad Waqas, Hussain Muhammad Abdullah, Waqar Ahmad, Ubeera Shahid, Usman Khurshid

**Affiliations:** 1 Research Society, Allama Iqbal Medical College, Lahore, Pakistan; 2 Department of Health, Government of Punjab, Pakistan

## Abstract

**Background:**

The success of the Global Polio Eradication Initiative was remarkable, but four countries - Afghanistan, Pakistan, India and Nigeria - never interrupted polio transmission. Pakistan reportedly achieved all milestones except interrupting virus transmission. The aim of the study was to establish valid and reliable estimate for: routine oral polio vaccine (OPV) coverage, logistics management and the quality of monitoring systems in health facilities, NIDs OPV coverage, the quality of NIDs service delivery in static centers and mobile teams, and to ultimately provide scientific evidence for tailoring future interventions.

**Methods:**

A cross-sectional study using lot quality assessment sampling was conducted in the District Nankana Sahib of Pakistan's Punjab province. Twenty primary health centers and their catchment areas were selected randomly as *'lots'*. The study involved the evaluation of 1080 children aged 12-23 months for routine OPV coverage, 20 health centers for logistics management and quality of monitoring systems, 420 households for NIDs OPV coverage, 20 static centers and 20 mobile teams for quality of NIDs service delivery. Study instruments were designed according to WHO guidelines.

**Results:**

Five out of twenty lots were rejected for unacceptably low routine immunization coverage. The validity of coverage was questionable to extent that all lots were rejected. Among the 54.1% who were able to present immunization cards, only 74.0% had valid immunization. Routine coverage was significantly associated with card availability and socioeconomic factors. The main reasons for routine immunization failure were absence of a vaccinator and unawareness of need for immunization. Health workers (96.9%) were a major source of information. All of the 20 lots were rejected for poor compliance in logistics management and quality of monitoring systems. Mean compliance score and compliance percentage for logistics management were 5.4 ± 2.0 (scale 0-9) and 59.4% while those for quality of monitoring systems were 3.3 ± 1.2 (scale 0-6) and 54.2%. The 15 out of 20 lots were rejected for unacceptably low NIDs coverage by finger-mark. All of the 20 lots were rejected for poor NIDs service delivery (mean compliance score = 11.7 ± 2.1 [scale 0-16]; compliance percentage = 72.8%).

**Conclusion:**

Low coverage, both routine and during NIDs, and poor quality of logistics management, monitoring systems and NIDs service delivery were highlighted as major constraints in polio eradication and these should be considered in prioritizing future strategies.

## Background

The success of the Global Polio Eradication Initiative (GPEI), launched after a World Health Assembly resolution of 1988, was remarkable and nearly 5 million children were protected from being paralyzed by 2003 [1,2]. About 10 billion doses of polio vaccine have been administered since 1988 on hundreds of national and sub-national immunization days (NIDs/SNIDs) at a cost of US$ 4.5 billion [3,4]. But four countries - Afghanistan, Pakistan, India and Nigeria - never interrupted wild polio virus (WPV) transmission and re-infection of 26 countries in 2006-07 raised questions about the feasibility of polio eradication. By the end of 2007, polio incidence had decreased by 35% and polio transmission had stopped in all but 6 re-infected countries [5], yet polio eradication seems illusory as endemic countries are missing targets and 1648 cases were reported worldwide in 2008 [6].

Pakistan adopted the Polio Eradication Initiative (PEI) within the Expanded Program on Immunization (EPI) in 1994 remarkably decreasing the number of cases to 32 by 2007 including 19 WPV type 1 and 13 WPV type 3 cases with virus found in 18 of 120 districts [2,6-8]. But polio resurged in 2008 infecting 49 districts with 118 cases including 81 WPV type 1 and 37 WPV type 3 cases - highest amongst the previous six years [2,6-8]. Pakistan reportedly achieved all targets set in the GPEI strategic plan but failed to interrupt virus transmission [5,9-11], probably due to sub-district coverage gaps, low routine coverage, operational weaknesses in the quality of services and large numbers of children missed during NIDs/SNIDs. The scenario calls for immediate action.

As health programs mature, large-scale surveys become less useful. Instead, data from small-scale studies are required to evaluate different aspects of a program [12]. The aim of this study, in one district of Pakistan's Punjab province, was to establish valid and reliable estimate for the effectiveness of polio eradication services and to ultimately provide scientific evidence for tailoring future interventions. Specific objectives were the evaluation of routine immunization services including oral polio vaccine (OPV) coverage and the characteristics of vaccinated and unvaccinated children, the evaluation of logistics management and the quality of monitoring systems, and the assessment of national immunization days (NIDs) including NIDs coverage and the quality of NIDs service delivery.

## Methods

### Design and Setting

A cross-sectional study using lot quality assessment techniques was conducted between March 02, 2009 and March 22, 2009 in District Nankana Sahib, located in the central Punjab, with about 100 health facilities and 2000 health personnel obliged to provide curative and preventive services to 1.8 million inhabitants of the district (administrative data, 2009). [Additional files 1 and 2]

### Sample

The sample was taken by the lot quality assessment sampling (LQAS) technique, using World Health Organization (WHO) guidelines, Brixton health software packages and Epi Info 6 [13-15]. A sample was separately taken for assessment of routine and NIDs coverage, logistics management and the quality of monitoring systems, and the quality of NIDs service delivery. For random selection of the lots of the catchment areas of primary health centers, primary health centers, static centers and mobile teams; a line listing of all the respective areas/facilities was obtained from the District Department of Health and the sample was randomly selected by using two-digit random number table generated by Epi Info 6. [Additional file 3]

### Sample for routine and NIDs coverage

For routine and NIDs coverage, 20 lots of the catchment areas of 20 primary health centers (PHCs) were randomly selected - having a population of 516,918 and approximate population in each lot was 20,000 - 30,000.

Study subjects for the routine OPV coverage assessment and the characteristics of routine immunization status included 1080 children aged 12 - 23 months from 20 lots. Statistical parameters included an accuracy level of ± 3, a confidence level of 95%, a total sample size of 1080 children, a total number of 20 lots, a lot sample size of 54 children, a low threshold level of 80%, a high threshold level of 99% and a decision value for lot rejection of >3 unimmunized children (P:99% = 100%, P:80% = 100%, Error = 0%).

Subjects for the NIDs coverage assessment were all children under-five present in 420 households in 20 lots. Statistical parameters included an accuracy level of ± 5, a confidence level of 95%, a total sample size of 420 households, a total number of 20 lots, a lot sample size of 21 households, a low threshold level of 90%, a high threshold level of 99% and a decision value for lot rejection of >0 households having one or more unimmunized children (P:99% = 81%, P:90% = 89%, Error = 30%).

### Sample for logistics management and the quality of monitoring systems

Out of 70 primary health centers (PHCs) in District Nankana Sahib, 20 randomly selected PHCs were evaluated for logistics management and the quality of monitoring systems in health facilities. With a low threshold level of 80% and a high threshold level of 99%, the decision value for lot rejection was >0 (P:99% = 86%, P:80% = 96%, Error = 18%), and based on the compliance score for logistics management and the quality of monitoring systems (scale = 0-15)), therefore acted as equivalent to the lot sample size.

### Sample for quality of NIDs service delivery

Static centers at each of the 20 randomly selected PHCs and one randomly selected mobile team from their catchment area were evaluated for the quality of NIDs service delivery. With a low threshold level of 80% and a high threshold level of 99%, the decision value for lot rejection was >0 (P:99% = 85%, P:80% = 97%, Error = 18%), and based on the compliance score for the quality of NIDs service delivery (scale = 0-16), therefore acted as equivalent to the lot sample size.

### Data Collection

Study instruments were designed according to WHO guidelines [13,16-18], pre-tested in the field and modified accordingly. [Additional file 4]

For evaluating routine OPV coverage, a standardized questionnaire consisting of sociodemographic information, immunization status and related characteristics was used in face-to-face interview in household settings. It took 10 minutes to fill out the questionnaire by response of child's caretaker (mother, if possible). Coverage was measured by "card" and by "card plus history". A child who received all immunizations required by the EPI schedule was considered fully immunized. Valid immunization meant an immunization given at the appropriate age (within six months), after an appropriate interval of time (according to EPI schedule) and recorded on an immunization card.

A list of all the villages/wards was obtained from the District Department of Health and three villages/wards in each lot were randomly selected as sampling point areas. As the villages/wards have different settlement designs in Pakistan, the mosque was considered the center of each village/ward. Interviewers went to the mosque, spun a bottle on level-ground, and the first household was selected by the direction in which the bottle pointed. The household was included in the sample if a child aged 12-23 months was present. Subsequent households were chosen by proximity. One child was seen from each household and in case of more than one children aged 12-23 months in a household, the youngest child was seen. In this way, 18 households were randomly selected from each sampling point area; therefore, 54 children were seen from each lot, making a total sample of 1080 children from the district.

For evaluating logistics management and the quality of monitoring systems, structured forms were filled out by observation and inspection of PHCs. Data were obtained from respective vaccination point (the center from where vaccine was collected) for PHCs with a non-functioning refrigerator.

For NIDs coverage, a standardized questionnaire was used in face-to-face interviews in household settings. It took 5 minutes to fill out the questionnaire. Coverage was measured by "finger-mark". A household was considered unimmunized if one or more of the children present were not immunized.

The sampling point areas selected for routine coverage were surveyed again during NIDs (March 16, 2009 - March 20, 2009). The 7 households were randomly selected from each sampling point area and all children in each household were seen. The 21 households were visited in this way from each lot, making a total sample of 420 households from the district.

For evaluating the quality of NIDs service delivery, structured forms were filled out by observation, inspection and exit interviews in static centers and mobile teams.

Verbal informed consent from respondents was deemed sufficient. Approval for the study was granted by the Ethical Review Board of Allama Iqbal Medical College, Lahore, Pakistan and the Department of Health, District Government Nankana Sahib, Punjab, Pakistan. Data were collected by medical students trained to carry out surveys and the entire process was monitored by a field coordinator. Health education for children's caretakers and technical assistance for health workers was also provided during the study.

### Data analysis

Data were entered and analyzed using SPSS version 17 (SPSS Inc. Chicago IL, United States: 2008). Weighted immunization coverage was calculated by using WHO guidelines for lot quality surveys [13]. For logistics management and the quality of monitoring systems in health facilities and the quality of NIDs service delivery, a weighted percentage was obtained. Each characteristic was equally weighed, a compliance score based on present/maintained characteristics was computed for each lot, and a mean compliance score was then obtained. A compliance percentage was calculated as sum of correct answers/total observations × 100. Bivariate analysis using the chi-square test was employed to independently correlate variables. Statistical significance was considered at P < 0.05.

## Results

### Routine Immunization Services

#### Routine OPV Coverage and characteristics of routine immunization status

Of 1080 children aged 12 - 23 months, 54.7% were male and 45.3% were female. Many children had illiterate parents (41.9%), 1 - 3 siblings (47.8%), a monthly family income of <5000 PKR (47.7%), poor housing (58.9%), and used public health facilities less than once a year (32.5%).

The 95% children were fully immunized by card plus history (5 out of 20 lots rejected because of having unacceptably low coverage of below 80%), 52.0% were fully immunized by card (all lots rejected because of having unacceptably low coverage of below 80%), and 42.0% had valid immunization (all lots were rejected because of having unacceptably low coverage of below 80%). [Table 1, Additional file 5] The drop-out rate between OPV I and OPV III was 4.0% by card plus history and 2.0% by card.

**Table 1:** Routine OPV III coverage among children aged 12-23 months (n = 1080)

Coverage	n	Weighted %	95% CI	Lots rejected (n = 20)
By card plus history	1030	95	93.9-96.5	5
By card	561	52	48.9-55.0	20
Valid	432	40	37.1-43.0	20

The 54.1% of respondents were able to present immunization cards and this was significantly associated with better education (P = 0.001), fewer siblings (P < 0.001) and good housing (P = 0.001). Valid immunization among children having cards was 74.0%. The 93.0% of children had received supplementary OPV doses during previous NIDs.

Immunization coverage was significantly higher (P < 0.001) among those having immunization cards (96.1%, 561/584) as compared to those assessed by history alone (94.6%, 469/496). Full immunization status was significantly associated with socioeconomic factors [Table 2]. The use of public health facilities was significantly associated with better education (P = 0.049).

**Table 2:** Predictors of immunization status against polio among children aged 12-23 months

Population circumstances	Total sample (n = 1080)	Fully immunized					
		
		Card plus history (n = 1030)		Card (n = 561)		Valid (n = 432)	
	
	n (%)	n (%)	**Sig**.	n (%)	**Sig**.	n (%)	**Sig**.
**Parental education**							
Illiterate	452 (41.9)	424 (93.8)	P = 0.054	202 (44.7)	P < 0.001	147 (32.5)	P < 0.001
Primary	321 (29.9)	313 (97.5)		190 (59.2)		149 (46.4)	
High School and above	307 (28.4)	293 (95.4)		169 (55.0)		136 (44.3)	
**Siblings**							
No	213 (19.7)	209 (98.1)	P = 0.036	131 (61.5)	P < 0.001	99 (46.5)	P < 0.001
1-3	516 (47.8)	493 (95.5)		277 (53.7)		222 (43.0)	
>3	351 (32.5)	327 (93.4)		153 (43.6)		111 (31.6)	
**Condition of house**							
Muddy/Mixed	636 (58.9)	599 (94.2)	P = 0.027	298 (46.9)	P < 0.001	228 (35.8)	P = 0.001
Cemented	444 (41.1)	431 (97.1)		263 (59.2)		204 (45.9)	
**Health seeking behavior**							
Once or more a month	297 (27.5)	287 (96.6)	P = 0.048	146 (49.2)	P = 0.253	119 (40.1)	P = 0.942
<Once a month	174 (16.1)	172 (98.9)		103 (59.2)		71 (40.8)	
Once in 6 month	171 (15.8)	161 (94.2)		86 (50.3)		68 (39.8)	
Once a Year	87 (8.1)	82 (94.3)		48 (55.3)		38 (43.7)	
<Once a Year	351 (32.5)	328 (93.4)		178 (50.7)		136 (38.7)	

Note: The table shows row percentages. Pearson chi-square test and Fisher's exact test are employed to obtain 2-sided significance (sig.)

Health workers were the source of immunization in all cases. Reasons cited for immunization failure were: vaccinator absent (n = 34), unaware of the need for immunization (n = 22), place of immunization too far (n = 15), time of immunization inconvenient (n = 15), unaware of the need to return for the next dose (n = 05), parents too busy (n = 04), fear of side effects (n = 04) and no faith in immunization (n = 02).

Health workers (96.9%) were the major source of information, including mosque announcements. Television (6.0%), brochures, posters and printed material (3.5%), religious leaders (2.3%), and others (1.9%) were additional sources.

#### Logistics management and the quality of monitoring systems in health facilities

Twenty PHCs, including 3 Rural Health Centers and 17 Basic Health Units, were evaluated. A medical officer was posted in 45.0% of health facilities. Syringes were being properly discarded in 85.0% of facilities. Essential medicines and equipment were available in 75.0% of facilities. The buildings and hygiene was good in 25.0% of facilities, and sign boards were properly placed in 50.0% of facilities.

All of the 20 lots were rejected for having poor compliance regarding logistics management and the quality of monitoring systems (less than 80%). [Table 3] The compliance scores for logistics management and the quality of monitoring systems for individual lots is given in Table 4. [Additional file 6]

**Table 3:** Quality of routine services regarding the Polio Eradication Initiative

Characteristics	Compliance score scale	Mean compliance score	Percentage compliance	Lots rejected (n = 20)
Logistics management in health facilities	0-9	5.4 ± 2.0	59.4	20
Quality of monitoring systems in health facilities	0-6	3.3 ± 1.2	54.2	20

**Table 4:** Compliance scores for individual lots regarding logistics management and the quality of monitoring systems (Scale 0-15)

**Lot No**.	Score	**Lot No**.	Score	**Lot No**.	Score	**Lot No**.	Score	**Lot No**.	Score
**1**	12	**5**	8	**9**	5	**13**	6	**17**	7
**2**	12	**6**	8	**10**	11	**14**	7	**18**	8
**3**	14	**7**	9	**11**	9	**15**	9	**19**	9
**4**	9	**8**	4	**12**	3	**16**	9	**20**	13

The mean compliance score for logistics management based on 9 characteristics was 5.4 ± 2.0, while the compliance percentage was 59.4%. For the quality of monitoring systems, the mean compliance score based on 6 characteristics was 3.3 ± 1.2, while the compliance percentage was 54.2%. [Table 3]

For each of the nine characteristics evaluated in logistics management in 20 PHCs, the quality status was acceptable for sufficient OPV stocks not close to or past expiry date during the past month (n = 20), and the availability of vehicles for vaccinators in working condition (n = 20). The quality status was poor for the functioning of refrigerators (n = 11), up-to-date refrigerator temperature records in the correct range (n = 14), vaccine storage in the correct part of the refrigerator (n = 15), the availability of frozen ice packs (n = 12), the availability and maintenance of cold boxes/vaccine carriers (n = 06), the availability and use of safety boxes (n = 07) and the availability of power generators (n = 02). [Table 5]

**Table 5:** Logistics management and the quality of monitoring systems in health facilities (n = 20)

Characteristics	n	Weighted %
**Logistics management**		
Sufficient OPV stock not close to or past expiry date during past month	20	95
Refrigerator functional and used for vaccine storage at health facility	11	65
Refrigerator temperature record up-to-date and in correct range (0-8°C)	14	80
OPV storage in correct part of refrigerator	15	85
Availability of frozen icepacks	12	70
Availability and maintenance of cold box/vaccine carriers	6	45
Availability of power generator	2	25
Availability and use of safety boxes at all vaccination sites	7	50
Functional vehicle for vaccinator	20	95
**Quality of monitoring systems**		
Visit of health facility by district managers in past three months and presence of their inspection notes	19	95
Availability and maintenance of EPI registers on desk	11	65
Up-to-date vaccine ledger for OPV	8	55
Availability of monthly facility staff meeting minutes held in past three months	3	30
Display of graphs/charts depicting health facility's PEI performance over time	12	70
Data accuracy (number of children receiving OPV III on EPI register vs. monthly EPI report matched)	12	70

For each of the six characteristics evaluated in the quality of monitoring systems, quality status was acceptable for visits by district managers to health facilities (n = 19). The quality status was poor for the maintenance of EPI registers (n = 11), the maintenance of vaccine ledgers (n = 08), the availability of meeting minutes (n = 03), the display of graphs/charts depicting the health facility's EPI/PEI performance over time (n = 12), and data accuracy in EPI registers (n = 12). [Table 5]

### National Immunization Days (NIDs)

#### NIDs OPV coverage

The total number of children under-five in sampled 420 households was 911. The number of children under-five per household was 2.2 and 86 of the children in sampled households were not available, leaving a total sample of 825 children. The NIDs OPV coverage by finger-mark was 92.0% and 15 out of 20 lots were rejected for having unacceptably low coverage of below 90%. [Table 6, Additional file 7]

**Table 6:** NIDs OPV coverage among children under-five (n = 825)

Coverage	n	Weighted %	95% CI	Lots rejected (n = 20)
By finger-mark	759	92	89.9-93.7	15

#### Quality of NIDs service delivery

NIDs static centers at 20 PHCs and one NIDs mobile team in the catchment area of each of the 20 PHCs was evaluated. The mean compliance score for the quality of NIDs service delivery based on 16 characteristics was 11.7 ± 2.1, while the compliance percentage was 72.8%. All of the 20 lots were rejected for poor compliance in the quality of NIDs service delivery (less than 80%). [Table 7] The compliance scores for NIDs service delivery for individual lots are given in Table 8. [Additional file 8]

**Table 7:** Quality of NIDs service delivery in static centers and mobile teams

Compliance score scale	0-16
Mean compliance score	11.7 ± 2.1
Percentage compliance	72.8
Lots rejected (n = 20)	20

**Table 8:** Compliance scores for individual lots regarding NIDs service delivery (Scale 0-16)

**Lot No**.	Score	**Lot No**.	Score	**Lot No**.	Score	**Lot No**.	Score	**Lot No**.	Score
**1**	13	**5**	12	**9**	12	**13**	10	**17**	5
**2**	13	**6**	13	**10**	14	**14**	11	**18**	12
**3**	14	**7**	10	**11**	11	**15**	12	**19**	11
**4**	13	**8**	9	**12**	13	**16**	14	**20**	11

For each of the 16 characteristics evaluated for the quality of NIDs service delivery in static centers and mobile teams, quality status was acceptable for immunization sessions being conducted in shade, in an orderly way without overcrowding (n = 20), and keeping unopened OPVs at adequate temperatures (n = 20) in static centers, and the covering of missed children on the same day (n = 19) in mobile teams. In static centers, quality status was poor for the availability of relevant staff - two health workers, including one lady health visitor who is trained for EPI/PEI activities at health facility (n = 16), correct filling of field attendance, vaccine distribution and tally sheets (n = 17), proper marking of immunization sites (n = 15), the availability of enough frozen ice/ice packs for the current session (n = 17), inquiries about the vaccination of children under 2 years (n = 15) and about acute flaccid paralysis in children under 15 years (n = 06), reminding the child's caretaker for the next round of NIDs (n = 0), explaining the end results of polio and the purpose of NIDs (n = 05), and adequate health workers' knowledge regarding vaccine vial monitors (VVMs) and unused OPV (n = 18). In mobile teams, the quality status was poor for correct storage of OPV - keeping vials dry and cold chain maintained as indicated by VVMs (n = 18), correct administration of OPV - 2 drops/child (n = 18), the presence of at least one female member (n = 16), and supervisory checking (n = 13). [Table 9]

**Table 9:** Quality of NIDs service delivery in static centers and mobile teams (n = 20)

Characteristics	n	Weighted %
**Static centers**		
Availability of relevant staff	16	90
Proper marking of immunization site	15	85
Conduction of immunization session in shade, orderly (clear flow of clients) and without overcrowding (<20 under-five children waiting)	20	95
Correct filling of field attendance, vaccine distribution and tally sheets	17	95
Unopened OPV kept at adequate temperature (0-8°C)	20	95
Availability of enough frozen ice packs/ice for current session	17	95
Adequate knowledge of health workers regarding VVMs and unused OPV	18	95
Inquiry about vaccination status of all children<2 years from child's caretaker	15	85
Inquiry about acute flaccid paralysis in children<15 years from child's caretaker	6	45
Reminding child's caretaker for next round of NIDs	0	0
Adequate knowledge of child's caretaker about end results of polio and purpose of NIDs	5	40
**Mobile teams**		
Correct storage of OPV (keeping vials dry and cold chain maintained as indicated by VVMs)	18	95
Correct administration of OPV (2 drops/child)	18	95
Covering of missed children on same day	19	95
Presence of at least one female member	16	90
Checking by supervisory staff	13	75

## Discussion and Conclusions

The historically used LQAS design is still an appealing technique for rapid evaluation of preventive programs, especially in rural areas of developing countries [19-23]. It is very efficient in determining the performance of individual subunits in a specific area [22,23]. It was observed that most health personnel were unaware of this technique and its effectiveness in local evaluations.

Five out of twenty lots were rejected for unacceptably low routine immunization coverage, while the validity of routine overage was questionable to the extent that all lots were rejected. Weakness in routine immunization is the main constraint in polio eradication [21]. A national immunization card program could significantly increase coverage and the validity of coverage [22,23]. Knowledge gaps underlie low compliance with vaccination schedules, and the quality of interaction between health workers and caretakers is essential to ensure compliance with vaccination schedules [24].

Nearly half of the children sampled could not present a card. Coverage was significantly higher among those having cards, consistent with previous studies [19,23,25,26]. Card availability and immunization status were significantly associated with socioeconomic factors, consistent with previous studies [23,26-28]. Health workers were the major source of immunization and information. Outreach services have been recommended as the most efficient way to increase coverage [27-29] and there is a need for interventions aimed at fostering the communication skills of health workers [30,31]. The main reasons for routine immunization failure were educational constraints and problems of accessibility and availability, in agreement with previous literature [19,21,23,32].

All 20 lots were rejected for poor compliance in logistics management and the quality of monitoring systems. Previous compliance studies have also indicated poor performance regarding cold chains and logistics [32-37]. The functioning of refrigerators was poor due to security and electricity problems. Maintenance of refrigerator temperatures within the correct range and vaccine storage were also poor, which can be attributed to the lack of technical skills among health workers and poor attitudes towards maintaining cold chain charts. Maintenance of frozen icepacks and cold box/vaccine carriers was poor. This merits necessary action because in case of electricity failure, it would be the only way to maintain cold chains, as power generators were not available at most of the facilities. Previous studies in India revealed vaccine carriers were non-compliant in about one-third facilities, and power failures were also indicated as major constraint [34,36]. Poor use of safety boxes indicates a lack of awareness about safety measures. A holistic rather than logistic approach should be used for vaccine safety [38].

The quality of monitoring systems and data was poor in all aspects, in agreement with previous studies [32,39-41]. This was attributed to the lack of supervision and knowledge among those who were responsible, consistent with previous studies [42]. There is a need for monitoring systems to be viewed with a broader perspective, not focusing only on technicalities but also on the support mechanisms [32,41,43]. In Pakistan, EPI/PEI records are not computerized; however, the feasibility of linked immunization database systems has been established elsewhere [44], therefore, computerizing EPI/PEI records and linking them with health management information systems already in place might improve data quality. The implications of a poor quality data system are reflected in the efficiency of health services. Previous experience has shown that significant improvements in data quality and monitoring systems can be made by data quality self-assessment (DQS) and the use of data for action [45-49]. This strategy is recommended in the Reaching Every District (RED) approach and Global Framework for Immunization Monitoring and Surveillance (GFIMS) by the World Health Organization [48-51].

The 15 out of 20 lots were rejected for unacceptably low NIDs coverage by finger-mark. Poor coverage during NIDs which had been designed to deliver supplementary OPV doses to all children has been indicated as an underlying factor for the continued transmission of polio [9,52].

All 20 lots were rejected for poor NIDs service delivery. Poor technical skills of health workers, logistic problems, poor planning, and deficient communication skills were highlighted as the main problems. Similar findings were previously reported and associated with increased risk of non-vaccination [36,53-56]. In particular, communication with the clients was very poor. Previous studies have revealed the importance of effective communication to improve the coverage [57,58]. Decisions have generally not been based on studies of populations' knowledge and attitudes about immunization. Had this datum been strategically used, interventions could have been more effective in reaching zero-dose children [54]. Poor planning and lack of technical skills accounted for service disruptions [59], and supportive supervision could significantly increase the performance of immunization sessions [36,46,60].

The study does have limitations. Since the status and validity of routine immunization obtained from cards was likely to be an underestimate, immunization by card plus history was included. It is an established practice to estimate coverage by maternal history [61,62]. Some characteristics evaluated in the study were of subjective nature that might be a limitation in itself. Central selection rather than the random walk method was followed in coverage estimation because of different settlement designs in villages/wards in Pakistan and that may be a limitation. The per lot error for NIDs coverage was high because of the low sample size as a higher sample was not logistically feasible; however, the error for overall coverage was not affected by that.

Although the study was conducted in a rural district of Pakistan's Punjab province, some of findings may be generalized to other areas with similar health system infrastructure, socio-cultural environments and topography. The findings suggest that LQAS studies could be conducted in other areas to assess performance on a sub-national level.

Short-comings revealed in polio eradication services are potentially important due to lack of similar studies in the region and the failure to achieve polio eradication despite continuous efforts by the global community over the past two decades. Following interventions are recommended:

• training of mid-level health managers in LQAS,

• administrative measures to improve routine coverage and its validity, including the appraisal of excellent performance, making flexible field plans to track missed children, initiating Immunization Card Crash Program, constituting law for birth registration with health centers, and improving linkage between preventive programs,

• consideration of socioeconomic status in prioritizing interventions especially focusing on the illiterate, those living in poor houses and having large families (indirect predictors of poverty) and those not seeking health care at a public health facility,

• focus on advocacy and communication including involving local communities in designing strategies, decentralizing resources, and training health workers in communication skills,

• up-gradating cold chain equipment including ensuring on-going availability and maintenance of equipment, addressing electricity and security problems, making a vaccination point at each PHC, and maintaining reserve stocks at district stores,

• capacity building of staff including establishing a training school for vaccinators, supportive supervision, and focused training programs held bi-annually for EPI/PEI staff at all levels, which should be mandatory for their promotion,

• computerizing EPI/PEI records integrated with district health management information system and encouraging realistic reporting,

• practically-oriented training and micro planning for NIDs and accessing the quality of NIDs service delivery, which is often overlooked in rapid campaign evaluations,

• measures to address the extreme shortage of public health professionals in Pakistan, which affects all preventive programs including the provision of incentives to medical graduates for joining public health/preventive medicine and establishing national public health services.

Further studies in the region to evaluate findings are suggested. Polio continues to be a public health problem and we need to have a better understanding of the factors involved in achieving polio eradication.

## Competing interests

The authors declare that they have no competing interests.

## Authors' contributions

All authors contributed significantly in all phases of the project in accordance with uniform requirements established by International Committee of Medical Journal Editors (ICMJE) and contributors who do not meet authorship criteria are listed in acknowledgements. All authors read and approved the final version of the manuscript.

## Pre-publication history

The pre-publication history for this paper can be accessed here:

http://www.biomedcentral.com/1471-2458/10/60/prepub

## Supplementary Material

Additional file 1 **Map of Pakistan's Punjab province showing District Nankana Sahib**. This is the map of Punjab province of Pakistan showing District Nankana Sahib Click here for file

Additional file 2 **Map of District Nankana Sahib showing lots**. This is the map of District Nankana Sahib showing the 20 selected lots where the study was conducted Click here for file

Additional file 3 **List of Lots selected from District Nankana Sahib and sampling point areas for coverage assessment**. This table enlists lots selected from District Nankana Sahib, their population and sampling point areas for coverage assessment Click here for file

Additional file 4 **Survey Instruments**. This document contains instruments used in the study Click here for file

Additional file 5 **Routine OPV coverage**. This table enlists detailed lot-vise data for routine OPV coverage Click here for file

Additional file 6 **Lot-vise detail of logistics management and quality of monitoring system**. This table enlists detailed lot-vise data for logistics management and quality of monitoring in health facilities Click here for file

Additional file 7 **National Immunization Days (NIDs) OPV coverage**. This table enlists detailed lot-vise data for NIDs OPV coverage Click here for file

Additional file 8 **Lot-vise detail of quality of services during NIDs**. This table enlists detailed lot-vise data for quality of services during NIDs Click here for file
