# Supplementary figures and images for: Are we doing enough? Evaluation of the Polio Eradication Initiative in a district of Pakistan's Punjab province: a LQAS study
Source: BMC Public Health. 2010 Feb 9;10:60. doi: 10.1186/1471-2458-10-60 (PMC2845105; doi:10.1186/1471-2458-10-60)

**FIGURE 1 – MAP OF PAKISTAN’S PUNJAB PROVINCE SHOWING DISTRICT NANKANA SAHIB**

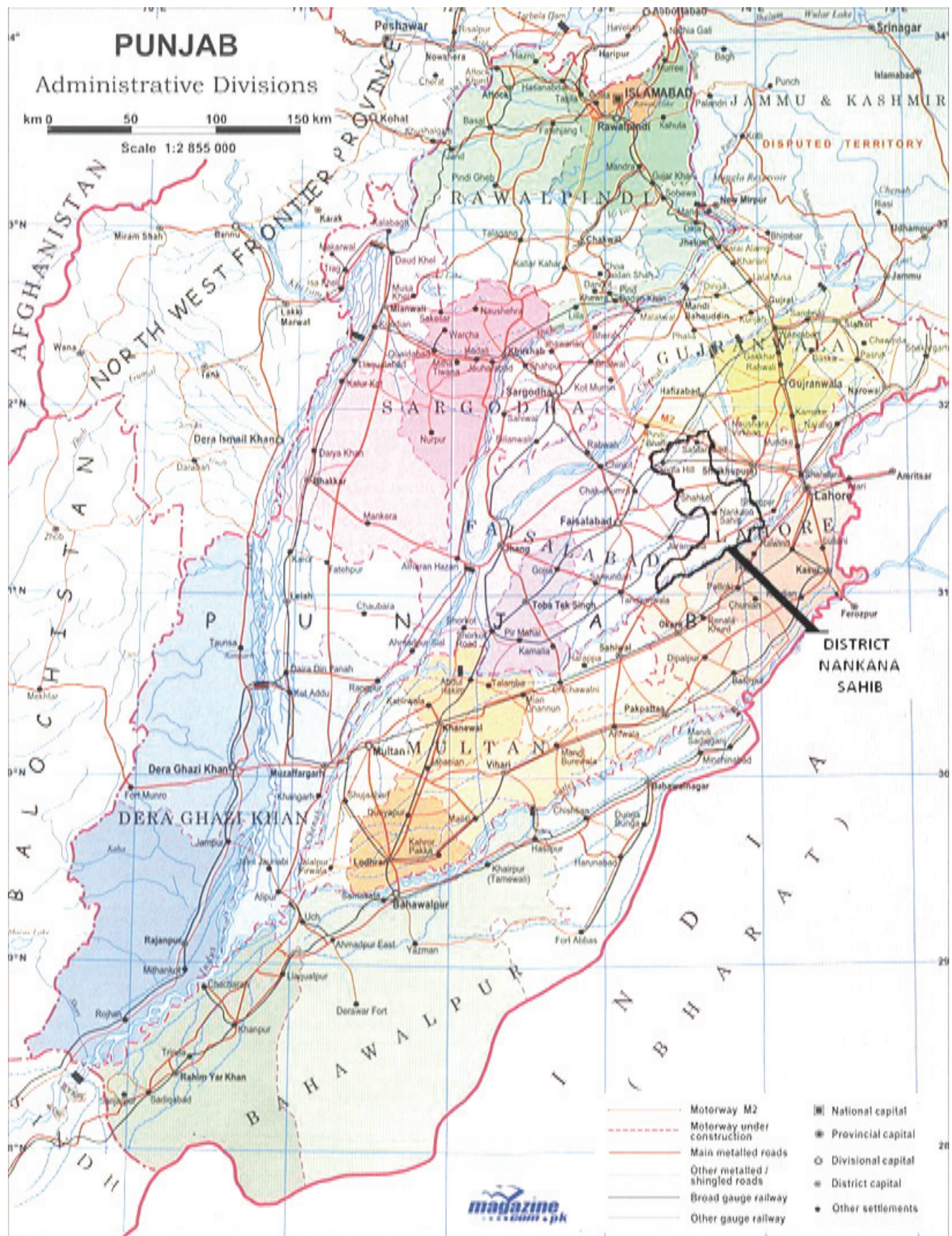

Supplement: Additional file 1 — Map of Pakistan's Punjab province showing District Nankana Sahib. This is the map of Punjab province of Pakistan showing District Nankana Sahib [file 1471-2458-10-60-S1.PDF]

**FIGURE 2 – MAP OF DISTRICT NANKANA SAHIB SHOWING LOT**

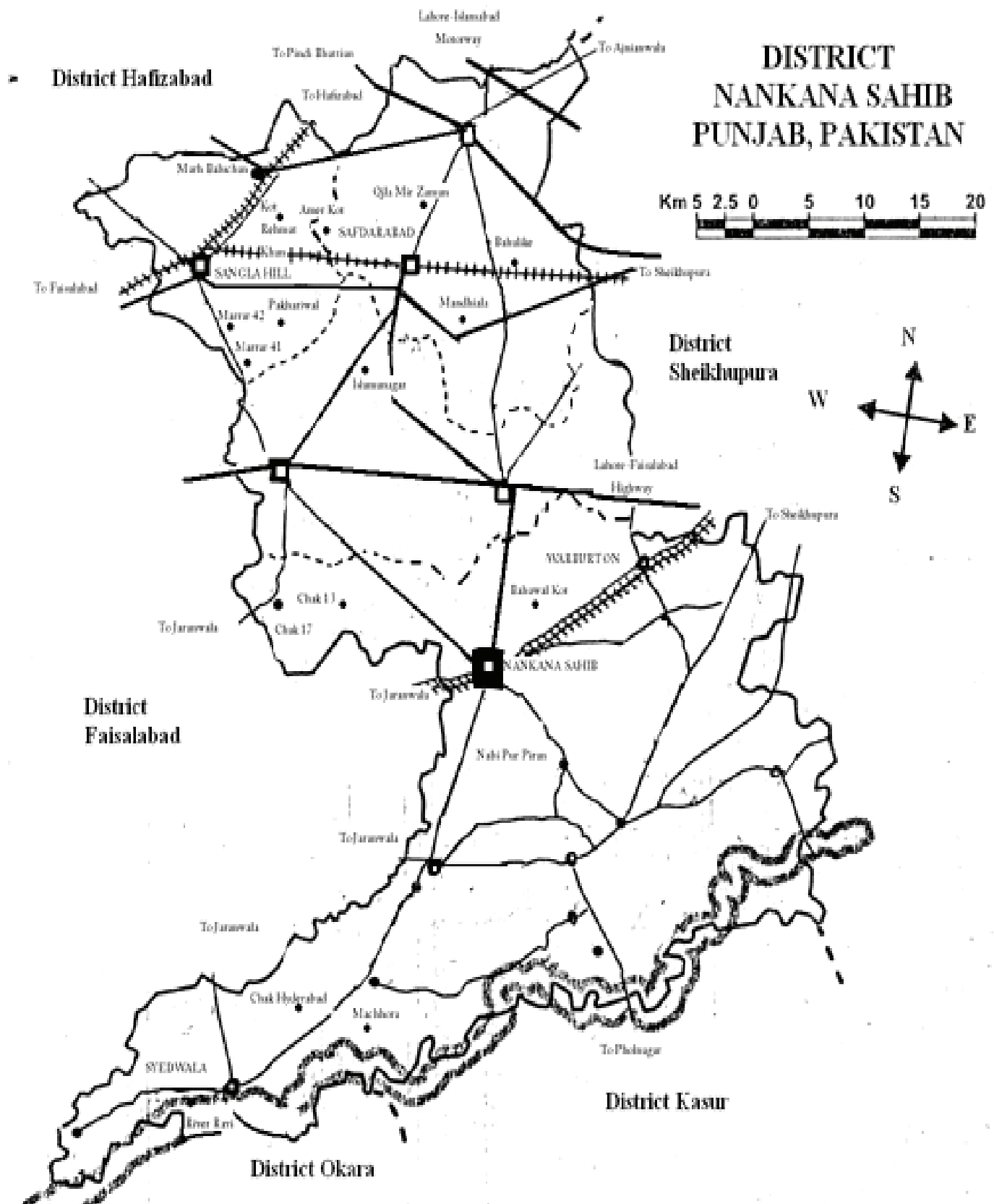

Supplement: Additional file 2 — Map of District Nankana Sahib showing lots. This is the map of District Nankana Sahib showing the 20 selected lots where the study was conducted [file 1471-2458-10-60-S2.PDF]
