# Supplementary material for: Are we doing enough? Evaluation of the Polio Eradication Initiative in a district of Pakistan's Punjab province: a LQAS study
Source: BMC Public Health. 2010 Feb 9;10:60. doi: 10.1186/1471-2458-10-60 (PMC2845105; doi:10.1186/1471-2458-10-60)
Supplement: Additional file 3 — List of Lots selected from District Nankana Sahib and sampling point areas for coverage assessment. This table enlists lots selected from District Nankana Sahib, their population and sampling point areas for coverage assessment [file 1471-2458-10-60-S3.PDF]

ANNEX 5 – **List of LOTS selected from District Nankana Sahib and sampling point areas for coverage assessment**

No.	LOT Name	Population	Sampling Points Areas for Immunization Coverage
01	RHC Syedwala	25512	Syedwala Naya, Syedwala Purana, Jhuggian Lahorian
02	RHU Warburton	29446	Warburton City Ward Number 1, 3, 6
03	RHU Sangla Hill	24914	Sangla Hill City UC SG-25 Ward Number 1, 3, 6
04	BHU Islamnagar	24461	Islamnagar, Nizampur Chelian Wala, Nizampur Mola Singh
05	BHU Youngsonabad	30468	Youngsanabad, Chak 5 Kalan, Chak 5 Khurd
06	BHU Qila Mir Zaman	30338	Qila Mir Zaman, Dera Ballan, Maryamabad
07	BHU Marrar Chak 42	19708	Marrar Chak 42, Kotla Khurd, Chathe Chak 26
08	BHU Amer Kot	39978	Amer Kot, Dera Jat Amer Kot, Ranger Nangel
09	BHU Bahalike	27594	Bahalike Station, Phulerwan, Grohanwala
10	BHU Chak 13 Randher	26526	Chak 13 Randher, Chak 10, Chak 11
11	BHU Marh Baluchan	17889	Marh Baluchan, Marh Sharqi, Marh Gharbi
12	BHU Pakhariwal	23561	Pakhariwal, Sathiali Kalan, Sathiali Khurd
13	BHU Chak Hyderabad	21874	Chak Hyderabad, Faridabad, Jameki
14	BHU Marrar Chak 41	19252	Tibbi Yar Di, Dairh, Radiana
15	BHU Kot Rehmat Khan	24468	Kot Rehmat Khan, Mahes Janobi, Mahes Shamali
16	BHU Nabi Pur Piran	23518	Nabi Pur Piran, Naliwala, Khepanwali
17	BHU Mandhiala	27545	Nawan Pind, Rahimabad, Gondlanwala
18	BHU Bahawalkot	32405	Bahawalkot, Bhagor, Pind Islamabad
19	BHU Machhora	23290	Machhora, Roopa Mahtam, Ahata Sneha
20	BHU Chak 17 Karial	24171	Chak 17 Karial, Chak 18, Chak 15
