# Supplementary material for: Are we doing enough? Evaluation of the Polio Eradication Initiative in a district of Pakistan's Punjab province: a LQAS study
Source: BMC Public Health. 2010 Feb 9;10:60. doi: 10.1186/1471-2458-10-60 (PMC2845105; doi:10.1186/1471-2458-10-60)
Supplement: Additional file 4 — Survey Instruments. This document contains instruments used in the study [file 1471-2458-10-60-S4.PDF]

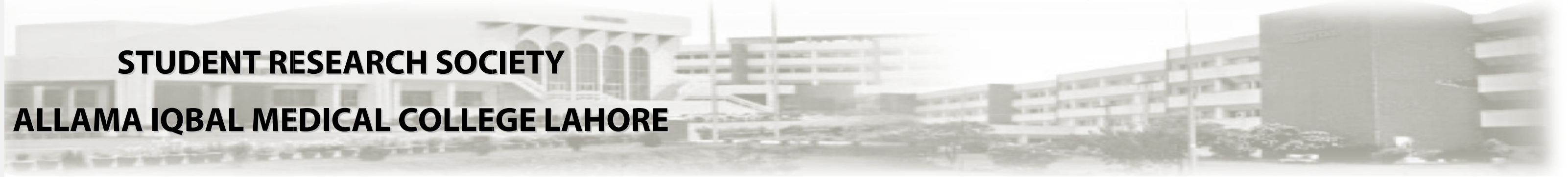

STUDENT RESEARCH SOCIETY

ALLAMA IQBAL MEDICAL COLLEGE LAHORE

SURVEY QUESTIONNAIRE

Information to be read to the respondent: **We are from Student Research Society of Allama Iqbal Medical College, Lahore and we are working on a project concerned with polio in children. We wish to learn about vaccination status of your child. We hope to understand your problems and the way to bring you the best preventive care. The information you provide will be used to improve polio eradication initiative in Pakistan. Your answers will not be released to anyone and will remain anonymous. Your participation is voluntary and you may choose to stop at any time. It will take 10 minutes, may I start now? Thank you for your assistance.** (*If permission is given start the interview*)

انٹرویو لینے سے پہلے مندرجہ ذیل معلومات فراہم کریں

ہمارا تعلق سٹوڈنٹ ریسرچ سوسائٹی علامہ اقبال میڈیکل کالج ،لاہور سے ہے اور ہم بچوں میں پولیو سے متعلق ایک منصوبے پر کام کر رہے ہیں۔ہم جاننا چاہتے ہیں کہ آپکے بچے کو پولیو کے قطرے پلائے گئے ہیں یا نہیں۔ہم امید کرتے ہیں کہ ہمیں آپکی ضروریات کو سمجھنے ،بچوں کو پولیو سے بچانے اور آپ تک علاج معالجے کی بہترین سہولیات پہنچانے میں مدد ملے گی۔آپ جو معلومات فراہم کریں گے اس سے پاکستان میں پولیو سے بچاؤ بہتر بنایا جا سکتا ہے۔آپ کے جوابات خفیہ رکھے جائیں گے۔آپ کا نام پر فارمہ پر نہیں لکھا جائے گا اور نہ ہی کسی اور ریکارڈ میں رکھا جائے گا۔آپکی شمولیت رضا کارانہ ہے اور آپ کسی بھی وقت انٹرویو میں شمولیت سے انکار کر سکتے ہیں۔اس انٹرویو پر 10 منٹ صرف ہوں گے۔کیا میں شروع کر سکتا/سکتی ہوں۔

آپ کے تعاون کا شکریہ (اگر اجازت مل جائے تو انٹرویو شروع کریں)

**Sociodemographic Profile**	Village/Ward:	Page Number:

Lot Name:	Lot Number:	Date of Visit:	Birth Date Range:

Child Number		1	2	3	4	5	6	7	8	9	10	11	12	13	14	15	16	17	18	Total
1. Parental Education	Illiterate																			
	Middle																			
	High school & Above																			
2. Works Outside	Father																			
	Mother																			
	Both																			
3. Siblings	No																			
	1-3																			
	>3																			
4. Income	Low (<5000PKR)																			
	Middle (5000-15000 PKR)																			
	HIGH (>15000 PKR)																			
5. Housing Condition	Muddy/Mixed																			
	Cemented																			
6. Public Health Facility Utilization	Once or more a month																			
	<Once a month																			
	Once in 6 month																			
	Once a Year																			
	<Once a Year																			

**SOCIODEMOGRAPHIC PROFILE**	VILLAGE/WARD:	PAGE NUMBER:

CHILD NUMBER		19	20	21	22	23	24	25	26	27	28	29	30	31	32	33	34	35	36	TOTAL
1. PARENTAL EDUCATION	ILLITERATE																			
	MIDDLE																			
	HIGH SCHOOL & ABOVE																			
2. WORKS OUTSIDE	FATHER																			
	MOTHER																			
	BOTH																			
3. SIBLINGS	NO																			
	1-3																			
	>3																			
4. INCOME	LOW (<5000PKR)																			
	MIDDLE (5000-15000 PKR)																			
	HIGH (>15000 PKR)																			
5. HOUSING CONDITION	MUDDY/MIXED																			
	CEMENTED																			
6. PUBLIC HEALTH FACILITY UTILIZATION	ONCE OR MORE A MONTH																			
	<ONCE A MONTH																			
	ONCE IN 6 MONTH																			
	ONCE A YEAR																			
	<ONCE A YEAR																			

**Sociodemographic Profile**	Village/Ward:	Page Number:

Child Number		37	38	39	40	41	42	43	44	45	46	47	48	49	50	51	52	53	54	Total
1. Parental Education	Illiterate																			
	Middle																			
	High school & Above																			
2. Works Outside	Father																			
	Mother																			
	Both																			
3. Siblings	No																			
	1-3																			
	>3																			
4. Income	Low (<5000PKR)																			
	Middle (5000-15000 PKR)																			
	HIGH (>15000 PKR)																			
5. Housing Condition	Muddy/Mixed																			
	Cemented																			
6. Public Health Facility Utilization	Once or more a month																			
	<Once a month																			
	Once in 6 month																			
	Once a Year																			
	<Once a Year																			

Supervisor: Name: ____________________________ Signature: _________________________

**Immunization Coverage Form**			Village/Ward:	Page Number:
Lot Name:	Lot Number:	Date of Visit:	Birth Date Range:	

Child Name																				Total		
																				Card	Card plus History	Immunization not given /not valid
Child Number		1	2	3	4	5	6	7	8	9	10	11	12	13	14	15	16	17	18			
Birth Date																						
Sex																						
Immunization Card	Yes/No																					
OPV I	Date/+/0																					
	Source																					
OPV II	Date/+/0																					
	Source																					
OPV III	Date/+/0																					
	Source																					
Supplementary Doses	Date/+/0																					
	Source																					

**Immunization Coverage Form**	Village/Ward:	Page Number:

Child Name																				Total		
																				Card	Card plus History	Immunization not given /not valid
Child Number		19	20	21	22	23	24	25	26	27	28	29	30	31	32	33	34	35	36			
Birth Date																						
Sex																						
Immunization Card	Yes/No																					
OPV I	Date/+/0																					
	Source																					
OPV II	Date/+/0																					
	Source																					
OPV III	Date/+/0																					
	Source																					
Supplementary Doses																						

**Immunization Coverage Form**	Village/Ward:	Page Number:

Child Name																				Total		
																				Card	Card plus History	Immunization not given /not valid
Child Number		37	38	39	40	41	42	43	44	45	46	47	48	49	50	51	52	53	54			
Birth Date																						
Sex																						
Immunization Card	Yes/No																					
OPV I	Date/+/0																					
	Source																					
OPV II	Date/+/0																					
	Source																					
OPV III	Date/+/0																					
	Source																					
Supplementary Doses																						

Date/+/0:
Date = copy date from immunization card
+ = Parents report history of immunization
0 = Immunization not given

Source:
OUT = Outreach
HC = Health Center
PRIV = Private

Supervisor:
Name: ___________________________

Signature: ________________________

**REASONS FOR IMMUNIZATION FAILURE**			VILLAGE/WARD:	PAGE NUMBER:
LOT NAME:	LOT NUMBER:	DATE OF VISIT:	BIRTH DATE RANGE:	

CHILD NUMBER		1	2	3	4	5	6	7	8	9	10	11	12	13	14	15	16	17	18	TOTAL
IMMUNIZATION STATUS	NOT IMMUNIZED																			
	PARTIALLY IMMUNIZED																			
	FULLY IMMUNIZED																			
UNAWARE OF NEED FOR IMMUNIZATION																				
UNAWARE OF NEED TO RETURN FOR NEXT DOSE																				
PLACE AND/OR TIME OF IMMUNIZATION UNKNOWN																				
FEAR OF SIDE EFFECTS																				
PLACE OF IMMUNIZATION TOO FAR																				
TIME OF IMMUNIZATION INCONVENIENT																				
VACCINATOR ABSENT																				
VACCINATOR TOLD CHILD WAS IMMUNIZED																				
VACCINE NOT AVAILABLE																				
PARENTS TOO BUSY																				
FAMILY PROBLEM INCLUDING MOTHER'S ILLNESS																				
CHILD ILL-NOT BROUGHT																				
CHILD ILL-BROUGHT BUT NOT VACCINATED																				
LONG WAITING TIME																				
NO FAITH IN IMMUNIZATION																				
RUMORS (SPECIFY)																				
OTHERS																				

REASONS FOR IMMUNIZATION FAILURE		VILLAGE/WARD:																	PAGE NUMBER:
CHILD NUMBER		19	20	21	22	23	24	25	26	27	28	29	30	31	32	33	34	35	36	TOTAL
IMMUNIZATION STATUS	NOT IMMUNIZED																			
	PARTIALLY IMMUNIZED																			
	FULLY IMMUNIZED																			
UNAWARE OF NEED FOR IMMUNIZATION																				
UNAWARE OF NEED TO RETURN FOR NEXT DOSE																				
PLACE AND/OR TIME OF IMMUNIZATION UNKNOWN																				
FEAR OF SIDE EFFECTS																				
PLACE OF IMMUNIZATION TOO FAR																				
TIME OF IMMUNIZATION INCONVENIENT																				
VACCINATOR ABSENT																				
VACCINATOR TOLD CHILD WAS IMMUNIZED																				
VACCINE NOT AVAILABLE																				
PARENTS TOO BUSY																				
FAMILY PROBLEM INCLUDING MOTHER'S ILLNESS																				
CHILD ILL-NOT BROUGHT																				
CHILD ILL-BROUGHT BUT NOT VACCINATED																				
LONG WAITING TIME																				
NO FAITH IN IMMUNIZATION																				
RUMORS (SPECIFY)																				
OTHERS																				

REASONS FOR IMMUNIZATION FAILURE		VILLAGE/WARD:																	PAGE NUMBER:	
CHILD NUMBER		37	38	39	40	41	42	43	44	45	46	47	48	49	50	51	52	53	54	TOTAL
IMMUNIZATION STATUS	NOT IMMUNIZED																			
	PARTIALLY IMMUNIZED																			
	FULLY IMMUNIZED																			
UNAWARE OF NEED FOR IMMUNIZATION																				
UNAWARE OF NEED TO RETURN FOR NEXT DOSE																				
PLACE AND/OR TIME OF IMMUNIZATION UNKNOWN																				
FEAR OF SIDE EFFECTS																				
PLACE OF IMMUNIZATION TOO FAR																				
TIME OF IMMUNIZATION INCONVENIENT																				
VACCINATOR ABSENT																				
VACCINATOR TOLD CHILD WAS IMMUNIZED																				
VACCINE NOT AVAILABLE																				
PARENTS TOO BUSY																				
FAMILY PROBLEM INCLUDING MOTHER'S ILLNESS																				
CHILD ILL-NOT BROUGHT																				
CHILD ILL-BROUGHT BUT NOT VACCINATED																				
LONG WAITING TIME																				
NO FAITH IN IMMUNIZATION																				
RUMORS (SPECIFY)																				
OTHERS																				

Supervisor: Name: ____________________________ Signature: _________________________

**Information Source**		Village/Ward:	Page Number:
Lot Name:	Lot Number:	Date of Visit:	Birth Date Range:

Child Number	1	2	3	4	5	6	7	8	9	10	11	12	13	14	15	16	17	18	Total
**From Where Do You Learned About OPV?**																			
TV																			
Radio																			
Newspapers and magazines																			
Brochures, posters and other printed materials																			
Health workers																			
Family, friends, neighbors and colleagues																			
Religious leaders																			
Teachers																			
Others																			
**Vaccinator Visits According to Plan (Y/N)**																			

**Information Source**	Village/Ward:	Page Number:

Child Number	19	20	21	22	23	24	25	26	27	28	29	30	31	32	33	34	35	36	Total
**From Where Do You Learned About OPV?**																			
TV																			
Radio																			
Newspapers and magazines																			
Brochures, posters and other printed materials																			
Health workers																			
Family, friends, neighbors and colleagues																			
Religious leaders																			
Teachers																			
Others																			

**Vaccinator Visits According to Plan (Y/N)**																		

**Information Source**	Village/Ward:	Page Number:

Child Number	37	38	39	40	41	42	43	44	45	46	47	48	49	50	51	52	53	54	Total
**From Where Do You Learned About OPV?**																			
TV																			
Radio																			
Newspapers and magazines																			
Brochures, posters and other printed materials																			
Health workers																			
Family, friends, neighbors and colleagues																			
Religious leaders																			
Teachers																			
Others																			
**Vaccinator Visits According to Plan (Y/N)**																			

Supervisor: Name: ____________________________ Signature: _________________________

**HEALTH FACILITY EVALUATION FORM**	DATE OF VISIT:
HEALTH FACILITY NAME:	SUPERVISOR'S NAME:
HF IN-CHARGE NAME:	SUPERVISOR'S SIGNATURE:

**A. LOGISTICS**	**YES**	**NO**	**COMMENTS**
1. The HF **has not** experienced any stock-out of OPV during past month?			
2. There is **no** OPV that is close to or past their expiry date			
3. The refrigerator temperature record is up-to-date and in the correct range			
4. The vaccines are stored in the correct part of the refrigerator			
5. Ice packs are available in frozen form			
6. Cold box / Vaccine carriers are maintained			
7. Generator is available			
8. Safety boxes are available and distributed to all vaccination sites			
9. Vehicle is available and in working condition			
**B. SUPERVISORY VISIT BY DISTRICT LEVEL SUPERVISORS**	**YES**	**NO**	**COMMENTS**
1. District manager(s) visited the health facility in the last three months			
2. Presence of Inspection notes of visit(s) in the last month by District manager(s) in the EPI Register/Inspection book maintained at the health facility			
3. All the visits planned by District Manager(s) in micro plans are performed			
**D. QUALITY OF MONITORING SYSTEM**	**YES**	**NO**	**COMMENTS**
1. The tally sheets and EPI register(s) are available on the desk and maintained			
2. The vaccine ledger is up to date for OPV vaccine			
3. The reports for past year can be easily retrieved			
4. Minutes of monthly facility staff meetings held in the last month are available			
5. Health facility performance was discussed during that meeting			
6. There is/are graph/charts on the wall showing the health facility's polio eradication performance over time (including catchment area map, target children, fixed/outreach teams, strategic indicators, drop-out rate etc.)			

E. DATA ACCURACY CHECK

**MONTH (SPECIFY)** *Count the number in the register and write down the figure, Transfer the number reported in the monthly report, If the monthly report is not available, then take the number from the district later*	**NUMBER FROM**		**NUMBERS MATCH**	
	**REGISTER**	**MONTHLY REPORT**	**YES**	**NO**
1. Number of children receiving OPV3 (EPI Daily Register(s) v EPI Report)				
2. Number of AFP Cases (OPD Register(s) v Report)				

SURVEY QUESTIONNAIRE

Information to be read to the respondent: **We are from Student Research Society of Allama Iqbal Medical College, Lahore and we are working on a project concerned with polio in children. We wish to learn about vaccination status of your child. We hope to understand your problems and the way to bring you the best preventive care. The information you provide will be used to improve polio eradication initiative in Pakistan. Your answers will not be released to anyone and will remain anonymous. Your participation is voluntary and you may choose to stop at any time. It will take 10 minutes, may I start now? Thank you for your assistance.** (*If permission is given start the interview*)

انٹرویو لینے سے پہلے مندرجہ ذیل معلومات فراہم کریں

ہمارا تعلق سٹوڈنٹ ریسرچ سوسائٹی علامہ اقبال میڈیکل کالج، لاہور سے ہے اور ہم بچوں میں پولیو سے متعلق ایک منصوبے پر کام کر رہے ہیں۔ ہم جاننا چاہتے ہیں کہ آپکے بچے کو پولیو کے قطرے پلائے گئے ہیں یا نہیں۔ ہم امید کرتے ہیں کہ ہمیں آپکی ضروریات کو سمجھنے، بچوں کو پولیو سے بچانے اور آپ تک علاج معالجے کی بہترین سہولیات پہنچانے میں مدد ملے گی۔ آپ جو معلومات فراہم کریں گے اس سے پاکستان میں پولیو سے بچاؤ بہتر بنایا جا سکتا ہے۔ آپ کے جوابات خفیہ رکھے جائیں گے۔ آپ کا نام پر فارمہ پر نہیں لکھا جائے گا اور نہ ہی کسی اور ریکارڈ میں رکھا جائے گا۔ آپکی شمولیت رضا کارانہ ہے اور آپ کسی بھی وقت انٹرویو میں شمولیت سے انکار کر سکتے ہیں۔ اس انٹرویو پر 10 منٹ صرف ہوں گے۔ کیا میں شروع کر سکتا/سکتی ہوں۔

آپ کے تعاون کا شکریہ (اگر اجازت مل جائے تو انٹرویو شروع کریں)

**NID Immunization Coverage Form**	Lot Name:	Lot Number:	Date of Visit:	Supervisor:

Village/Ward																						
Household Address																						
Household Number		1	2	3	4	5	6	7	8	9	10	11	12	13	14	15	16	17	18	19	20	21
Total Children Under-5																						
Children Immunized	By History																					
	By Finger Mark																					
Reasons For Immunization Failure	Children Not Available																					
	Team Absent																					
	Caretakers Refused																					
	Other																					
Wall Chalking was Correct	Yes																					
	No																					
Comments																						

NID Evaluation Form	Date of Visit:
Health Facility Name:	Supervisor's Name:
HF In-Charge Name:	Supervisor's Signature:

A. NID Static Center at Health Facility	Yes	No	Comments
Sufficient number of staff is present			
The site is well marked			
Immunizations are given in shade			
Waiting area is shaded			
The session is orderly (clear flow of clients)			
The session is without overcrowding (<20 under-5 children waiting)			
NID tally sheet is filled in correctly			
Care taker is welcomed			
Record keeper asks the age of all children			
Record keeper asks the vaccination status of all children under 2 yr age			
Record keeper asks about any acute lameness in children < 15 yrs			
Care taker is reminded when to come back for next round			
Health workers were courteous			
Care taker is thanked for coming			
Care taker know which vaccine was given			
Care taker can explain end results of Polio and purpose of NIDs			
OPV is being given correctly (2 drops/child)			
OPV supply is sufficient for this round of NIDs			
Unopened OPV being kept on or in ice packs (between 0-8 C)			
Health workers know the instructions for unused NID OPV			
The VVMs indicate that cold chain has been maintained			
Health workers know how to interpret VVMs			
There are enough frozen ice packs/ice for today's session			
Vaccine carrier and ice packs were available			
Field attendance and vaccine distribution sheets available and filled correctly			
Responsible health staff (Please Mention)			
**A. Mobile Team in Health facility Catchment Area**	**Yes**	**No**	**Comments**
The team members received training			
The team can be identified easily (badge, cap etc)			
OPV vials are dry			
VVM indicate that cold chain has been maintained			
OPV is being given correctly (Child number match with the vials used)			
Defaulter children are being noted			
The team has the area map/details			
The team can explain their assigned area			
The team covers the defaulter/missed children on the same day			
The team have at least one female member			
The team has been checked by supervisory staff			
