# Supplementary material for: Are we doing enough? Evaluation of the Polio Eradication Initiative in a district of Pakistan's Punjab province: a LQAS study
Source: BMC Public Health. 2010 Feb 9;10:60. doi: 10.1186/1471-2458-10-60 (PMC2845105; doi:10.1186/1471-2458-10-60)
Supplement: Additional file 5 — Routine OPV coverage. This table enlists detailed lot-vise data for routine OPV coverage [file 1471-2458-10-60-S5.PDF]

ANNEX 1- **ROUTINE OPV IMMUNIZATION COVERAGE**

LOT QUALITY ASSESSMENT

Lot No.	Lot Name	OPV Immunization Coverage (n=54)												Fully Immunized (n=54)		
		OPV 1				OPV 2				OPV 3						
		Card	Card plus History	Not Valid	Not Given	Card	Card plus History	Not Valid	Not Given	Card	Card plus History	Not Valid	Not Given	Card	Valid	Card plus History
**01**	**RHC Syedwala**	26	54	29	0	22	49	36	5	19	45	42	9	**19**	**12**	**45**
**02**	**RHC Warburton**	31	54	25	0	31	54	28	0	31	52	32	2	**31**	**22**	**52**
**03**	**RHC Sangla Hill**	42	54	13	0	42	54	13	0	41	51	18	1	**41**	**36**	**53**
**04**	**BHU Islamnagar**	30	54	32	0	30	54	33	0	30	52	38	2	**30**	**16**	**52**
**05**	**BHU Youngsonabad**	26	54	29	0	26	53	29	1	26	50	34	4	**26**	**20**	**50**
**06**	**BHU Qila Mir Zaman**	22	54	32	0	22	54	32	0	22	53	33	1	**22**	**21**	**53**
**07**	**BHU Marrar Chak 42**	39	53	15	1	38	52	17	2	37	51	21	3	**37**	**33**	**51**
**08**	**BHU Amer Kot**	26	54	30	0	26	54	34	0	26	50	36	4	**26**	**18**	**50**

**09**	**BHU Bahalike**	24	54	38	0	24	54	38	0	22	52	38	2	**22**	**16**	**52**
**10**	**BHU Chak 13 Randher**	41	54	18	0	41	54	19	0	40	53	22	1	**40**	**32**	**53**
**11**	**BHU Marh Baluchan**	31	54	26	0	30	53	28	1	29	52	29	2	**29**	**25**	**52**
**12**	**BHU Pakhariwal**	30	54	26	0	30	54	26	0	29	52	32	2	**29**	**22**	**52**
**13**	**BHU Chak Hyderabad**	24	54	30	0	24	54	31	0	23	53	35	1	**23**	**19**	**53**
**14**	**BHU Marrar Chak 41**	27	53	29	1	27	53	33	1	25	49	37	5	**25**	**17**	**49**
**15**	**BHU Kot Rehmat Khan**	26	54	28	0	26	54	28	0	26	53	31	1	**26**	**23**	**53**
**16**	**BHU Nabi Pur Piran**	30	54	29	0	30	54	32	0	30	50	32	4	**30**	**22**	**50**
**17**	**BHU Mandhiala**	27	54	32	0	27	54	34	0	27	53	34	1	**27**	**20**	**53**
**18**	**BHU Bahawalkot**	21	54	35	0	20	53	36	1	19	51	38	3	**19**	**16**	**51**
**19**	**BHU Machhora**	17	53	40	1	18	54	44	0	17	53	46	1	**17**	**8**	**53**
**20**	**BHU Chak 17 Karial**	42	53	16	1	42	53	20	1	42	53	20	1	**42**	**34**	**53**

Note: Decision value for lot rejection >3 unimmunized children

ANNEX 1- **ROUTINE OPV IMMUNIZATION COVERAGE (Continued)**

AGGREGATED DATA FOR LOT QUALITY COVERAGE SURVEY

Lot No.	Lot Name	Lot Total Population	Lot Target Population (Children aged 12-23 months – 3.397%)	Weight	Lot Sample Size	Number Immunized			Proportion Immunized		
						Card	Valid	Card plus History	Card	Valid	Card plus History
01	RHC Syedwala	25512	867	0.049	54	19	12	45	0.352	0.222	0.833
02	RHC Warburton	29446	1000	0.057	54	31	22	52	0.574	0.407	0.963
03	RHC Sangla Hill	24914	846	0.048	54	41	36	53	0.759	0.667	0.982
04	BHU Islamnagar	24461	831	0.047	54/-+	30	16	52	0.556	0.296	0.963
05	BHU Youngsonabad	30468	1034	0.059	54	26	20	50	0.482	0.370	0.926
06	BHU Qila Mir Zaman	30338	1031	0.059	54	22	21	53	0.407	0.389	0.982
07	BHU Marrar Chak 42	19708	669	0.038	54	37	33	51	0.685	0.611	0.944
08	BHU Amer Kot	39978	1358	0.077	54	26	18	50	0.482	0.333	0.926
09	BHU Bahalike	27594	937	0.053	54	22	16	52	0.407	0.296	0.963

10	BHU Chak 13 Randher	26526	901	0.051	54	40	32	53	0.741	0.596	0.982
11	BHU Marh Baluchan	17889	608	0.035	54	29	25	52	0.537	0.463	0.963
12	BHU Pakhariwal	23561	800	0.046	54	29	22	52	0.537	0.407	0.963
13	BHU Chak Hyderabad	21874	743	0.042	54	23	19	53	0.426	0.352	0.982
14	BHU Marrar Chak 41	19252	654	0.037	54	25	17	49	0.463	0.315	0.907
15	BHU Kot Rehmat Khan	24468	831	0.047	54	26	23	53	0.482	0.426	0.982
16	BHU Nabi Pur Piran	23518	799	0.046	54	30	22	50	0.556	0.407	0.926
17	BHU Mandhiala	27545	936	0.053	54	27	20	53	0.5	0.370	0.982
18	BHU Bahawalkot	32405	1101	0.063	54	19	16	51	0.352	0.296	0.944
19	BHU Machhora	23290	791	0.045	54	17	8	53	0.315	0.148	0.982
20	BHU Chak 17 Karial	24171	821	0.047	54	42	34	53	0.778	0.639	0.982
TOTAL		516918	17558	-	1080	561	432	1030	0.515	0.397	0.953
Weighted OPV-III Immunization Coverage in District Nankana Sahib									52%	40%	95%
