# Supplementary material for: Are we doing enough? Evaluation of the Polio Eradication Initiative in a district of Pakistan's Punjab province: a LQAS study
Source: BMC Public Health. 2010 Feb 9;10:60. doi: 10.1186/1471-2458-10-60 (PMC2845105; doi:10.1186/1471-2458-10-60)
Supplement: Additional file 6 — Lot-vise detail of logistics management and quality of monitoring system. This table enlists detailed lot-vise data for logistics management and quality of monitoring in health facilities [file 1471-2458-10-60-S6.PDF]

ANNEX 2 – **FACILITY-VISE DETAIL OF LOGISTICS MANAGEMENT AND QUALITY OF MONITORING SYSTEM**

Characteristics	Primary Health Centers									
	RHC Syedwala	RHC Warburton	RHC Sangla Hill	BHU Islamnagar	BHU Youngson-abad	BHU Qila Mir Zaman	BHU Chak 42 Marrar	BHU Amer Kot	BHU Bahalike	BHU Chak 13 Randher
**Logistics Management**										
Sufficient OPV stock not close to or past expiry date during past month	Yes	Yes	Yes	Yes	Yes	Yes	Yes	Yes	Yes	Yes
Refrigerator functional and used for vaccine storage at health facility	Yes	Yes	Yes	-	Yes	-	Yes	-	-	Yes
Refrigerator temperature record up-to-date and in correct range (0-8 °C)	Yes	Yes	Yes	Yes	Yes	Yes	Yes	-	-	Yes
OPV storage in correct part of refrigerator	-	Yes	Yes	Yes	Yes	Yes	Yes	-	-	Yes
Availability of frozen icepacks	Yes	Yes	Yes	Yes	Yes	Yes	-	-	-	Yes
Availability and maintenance of cold box/vaccine carriers	Yes	Yes	Yes	-	-	Yes	-	-	-	-
Availability of power generator	Yes	-	Yes	-	-	-	-	-	-	-
Availability and use of safety boxes at all vaccination sites	-	Yes	Yes	-	-	-	-	-	-	Yes
Functional vehicle for vaccinator	Yes	Yes	Yes	Yes	Yes	Yes	Yes	Yes	Yes	Yes
**Quality of Monitoring System**										
Visit of health facility by district managers in past three months and presence of their inspection notes	Yes	Yes	Yes	Yes	Yes	Yes	Yes	Yes	Yes	Yes
Availability and maintenance of EPI registers on desk	Yes	Yes	Yes	Yes	-	-	Yes	-	Yes	Yes
Up-to-date vaccine ledger for OPV	Yes	-	Yes	-	-	-	-	-	-	Yes
Availability of monthly facility staff meeting minutes held in past three months	-	-	-	Yes	-	-	-	-	-	-
Display of graphs/charts depicting health facility's PEI performance over time	Yes	Yes	Yes	Yes	-	Yes	Yes	Yes	Yes	-
Data accuracy (number of children receiving OPV III on EPI register vs. monthly EPI report matched)	Yes	Yes	Yes	-	Yes	-	Yes	-	-	Yes

ANNEX 2 –**FACILITY-VISE DETAIL OF LOGISTICS MANAGEMENT AND QUALITY OF MONITORING SYSTEM (Continued)**

Characteristics	Primary Health Centers									
	BHU Marh Baluchan	BHU Pakhariwal	BHU Chak Hyderabad	BHU Chak 41 Marrar	BHU Kot Rehmat	BHU Nabi Pur Piran	BHU Mandhiala	BHU Bahawal Kot	BHU Machora	BHU Chak 17 Karial
**Logistics Management**										
Sufficient OPV stock not close to or past expiry date during past month	Yes	Yes	Yes	Yes	Yes	Yes	Yes	Yes	Yes	Yes
Refrigerator functional and used for vaccine storage at health facility	Yes	-	-	Yes	-	-	-	Yes	Yes	Yes
Refrigerator temperature record up-to-date and in correct range (0-8 °C)	Yes	-	Yes	-	Yes	Yes	-	-	Yes	Yes
OPV storage in correct part of refrigerator	Yes	-	-	Yes	Yes	Yes	Yes	Yes	Yes	Yes
Availability of frozen icepacks	-	-	Yes	-	-	Yes	Yes	-	Yes	Yes
Availability and maintenance of cold box/vaccine carriers	-	-	-	-	-	-	Yes	-	-	Yes
Availability of power generator	-	-	-	-	-	-	-	-	-	-
Availability and use of safety boxes at all vaccination sites	-	-	-	-	Yes	Yes	-	Yes	-	Yes
Functional vehicle for vaccinator	Yes	Yes	Yes	Yes	Yes	Yes	Yes	Yes	Yes	Yes
**Quality of Monitoring System**										
Visit of health facility by district managers in past three months and presence of their inspection notes	Yes	-	Yes	Yes	Yes	Yes	Yes	Yes	Yes	Yes
Availability and maintenance of EPI registers on desk	Yes	-	-	Yes	-	-	Yes	-	-	Yes
Up-to-date vaccine ledger for OPV	Yes	-	Yes	-	Yes	Yes	-	-	-	Yes
Availability of monthly facility staff meeting minutes held in past three months	-	-	-	-	-	-	-	Yes	-	Yes
Display of graphs/charts depicting health facility's PEI performance over time	Yes	-	-	Yes	Yes	-	-	-	Yes	-
Data accuracy (number of children receiving OPV III on EPI register vs. monthly EPI report matched)	-	Yes	-	-	Yes	Yes	-	Yes	Yes	Yes
