# Supplementary material for: Are we doing enough? Evaluation of the Polio Eradication Initiative in a district of Pakistan's Punjab province: a LQAS study
Source: BMC Public Health. 2010 Feb 9;10:60. doi: 10.1186/1471-2458-10-60 (PMC2845105; doi:10.1186/1471-2458-10-60)
Supplement: Additional file 7 — National Immunization Days (NIDs) OPV coverage. This table enlists detailed lot-vise data for NIDs OPV coverage [file 1471-2458-10-60-S7.PDF]

ANNEX 3 - **NATIONAL IMMUNIZATION DAYS (NIDs) OPV COVERAGE**

LOT QUALITY ASSESSMENT

Lot No.	Lot Name	OPV Immunization			Households Immunized (n=21)
		Total children under 5	Children not present	Immunized (By finger mark)	Immunized (By finger mark)
**01**	**RHC Syedwala**	41	8	31	**20**
**02**	**RHC Warburton**	43	2	20	**13**
**03**	**RHC Sangla Hill**	37	6	30	**20**
**04**	**BHU Islamnagar**	40	11	27	**19**
**05**	**BHU Youngsonabad**	46	0	46	**21**
**06**	**BHU Qila Mir Zaman**	46	0	46	**21**
**07**	**BHU Marrar Chak 42**	44	11	29	**18**
**08**	**BHU Amer Kot**	61	1	59	**20**

**09**	**BHU Bahalike**	53	7	46	**21**
**10**	**BHU Chak 13 Randher**	47	4	43	**21**
**11**	**BHU Marh Baluchan**	45	0	45	**21**
**12**	**BHU Pakhariwal**	40	8	30	**20**
**13**	**BHU Chak Hyderabad**	44	5	35	**19**
**14**	**BHU Marrar Chak 41**	49	1	38	**12**
**15**	**BHU Kot Rehmat Khan**	44	2	36	**16**
**16**	**BHU Nabi Pur Piran**	45	1	43	**20**
**17**	**BHU Mandhiala**	40	7	31	**20**
**18**	**BHU Bahawalkot**	54	2	51	**20**
**19**	**BHU Machhora**	44	4	36	**18**
**20**	**BHU Chak 17 Karial**	48	6	37	**19**

Note: Immunized household means a household where all the children present were immunized. A household was considered unimmunized if one or more children were not immunized. Decision value for lot rejection >0 household having one or more unimmunized children

ANNEX 3 - **NATIONAL IMMUNIZATION DAYS (NIDs) OPV COVERAGE (Continued)**									
**AGGREGATED DATA FOR LOT QUALITY COVERAGE SURVEY**									
**Lot No.**	**Lot Name**	**Lot Total Population**	**Lot Target Population (Children under 5 yrs of age – 16%)**	**Weight**	**Lot Sample Size (Total House holds)**	**Total children under 5**	**Children not present**	**Immunized children (By Finger mark)**	
								**Number**	**Proportion**
01	RHC Syedwala	25512	4082	0.049	21	41	8	31	0.939
02	RHC Warburton	29446	4711	0.057	21	43	2	20	0.488
03	RHC Sangla Hill	24914	3986	0.048	21	37	6	30	0.968
04	BHU Islamnagar	24461	3914	0.047	21	40	11	27	0.931
05	BHU Youngsonabad	30468	4875	0.059	21	46	0	46	1
06	BHU Qila Mir Zaman	30338	4854	0.059	21	46	0	46	1
07	BHU Marrar Chak 42	19708	3153	0.038	21	44	11	29	0.879
08	BHU Amer Kot	39978	6396	0.077	21	61	1	59	0.983
09	BHU Bahalike	27594	4415	0.053	21	53	7	46	1
10	BHU Chak 13 Randher	26526	4244	0.051	21	47	4	43	1
11	BHU Marh Baluchan	17889	2862	0.035	21	45	0	45	1

12	BHU Pakhariwal	23561	3770	0.046	21	40	8	30	0.938
13	BHU Chak Hyderabad	21874	3500	0.042	21	44	5	35	0.897
14	BHU Marrar Chak 41	19252	3080	0.037	21	49	1	38	0.792
15	BHU Kot Rehmat Khan	24468	3915	0.047	21	44	2	36	0.857
16	BHU Nabi Pur Piran	23518	3763	0.045	21	45	1	43	0.977
17	BHU Mandhiala	27545	4407	0.053	21	40	7	31	0.939
18	BHU Bahawalkot	32405	5185	0.063	21	54	2	51	0.981
19	BHU Machhora	23290	3726	0.045	21	44	4	36	0.900
20	BHU Chak 17 Karial	24171	3867	0.047	21	48	6	37	0.881
TOTAL		516918	82705	-	420	911	86	759	0.919
Weighted NIDs OPV Coverage in District Nankana Sahib									92%
