# Supplementary material for: Are we doing enough? Evaluation of the Polio Eradication Initiative in a district of Pakistan's Punjab province: a LQAS study
Source: BMC Public Health. 2010 Feb 9;10:60. doi: 10.1186/1471-2458-10-60 (PMC2845105; doi:10.1186/1471-2458-10-60)
Supplement: Additional file 8 — Lot-vise detail of quality of services during NIDs. This table enlists detailed lot-vise data for quality of services during NIDs [file 1471-2458-10-60-S8.PDF]

ANNEX 4 – **FACILITY-VISE DETAIL OF LOGISTICS MANAGEMENT AND QUALITY OF SERVICES DURING NIDS**

Characteristics	Primary Health Centers									
	RHC Syedwala	RHC Warburton	RHC Sangla Hill	BHU Islamnagar	BHU Youngson-abad	BHU Qila Mir Zaman	BHU Chak 42 Marrar	BHU Amer Kot	BHU Bahalike	BHU Chak 13 Randher
**Static Centers**										
Availability of relevant staff	Yes	Yes	Yes	Yes	Yes	Yes	Yes	-	Yes	Yes
Proper marking of immunization site	Yes	Yes	Yes	Yes	Yes	Yes	-	-	Yes	Yes
Conduction of immunization session in shade, orderly (clear flow of clients) and without overcrowding (<20 under-five children waiting)	Yes	Yes	Yes	Yes	Yes	Yes	Yes	Yes	Yes	Yes
Correct filling of field attendance, vaccine distribution and tally sheets	Yes	Yes	Yes	Yes	Yes	Yes	Yes	Yes	-	Yes
Unopened OPV kept at adequate temperature (0-8 °C)	Yes	Yes	Yes	Yes	Yes	Yes	Yes	Yes	Yes	Yes
Availability of enough frozen ice packs/ice for current session	Yes	-	Yes	Yes	-	Yes	Yes	Yes	-	Yes
Adequate knowledge of health workers regarding VVMs and unused OPV	Yes	Yes	Yes	Yes	Yes	Yes	-	Yes	Yes	Yes
Inquiry about vaccination status of all children<2 years from child's caretaker	Yes	Yes	Yes	Yes	Yes	Yes	Yes	Yes	Yes	Yes
Inquiry about acute flaccid paralysis in children<15 years from child's caretaker	-	-	Yes	-	-	Yes	-	-	Yes	Yes
Reminding child's caretaker for next round of NIDs	-	-	-	-	-	-	-	-	-	-
Adequate knowledge of child's caretaker about end results of polio and purpose of NIDs	-	Yes	Yes	-	Yes	-	-	-	Yes	-
**Mobile Teams**										
Correct storage of OPV (keeping vials dry and cold chain maintained as indicated by VVMs)	Yes	Yes	Yes	Yes	Yes	Yes	Yes	-	Yes	Yes
Correct administration of OPV (2 drops/child)	Yes	Yes	Yes	Yes	Yes	Yes	Yes	Yes	Yes	Yes
Covering of missed children on same day	Yes	Yes	Yes	Yes	Yes	Yes	-	Yes	Yes	Yes
Presence of at least one female member	Yes	Yes	Yes	Yes	-	Yes	Yes	Yes	-	Yes
Checking by supervisory staff	Yes	Yes	-	Yes	Yes	-	Yes	-	Yes	Yes

ANNEX 4 – **FACILITY-VISE DETAIL OF LOGISTICS MANAGEMENT AND QUALITY OF SERVICES DURING NIDS (Continued)**

Characteristics	Primary Health Centers									
	BHU Marh Baluchan	BHU Pakhariwal	BHU Chak Hyderabad	BHU Chak 41 Marrar	BHU Kot Rehmat Khan	BHU Nabi Pur Piran	BHU Mandhiala	BHU Bahawal Kot	BHU Machora	BHU Chak 17 Karial
**Static Centers**										
Availability of relevant staff	Yes	Yes	-	Yes	Yes	Yes	-	Yes	-	Yes
Proper marking of immunization site	Yes	-	Yes	-	Yes	Yes	-	Yes	Yes	Yes
Conduction of immunization session in shade, orderly (clear flow of clients) and without overcrowding (<20 under-five children waiting)	Yes	Yes	Yes	Yes	Yes	Yes	Yes	Yes	Yes	Yes
Correct filling of field attendance, vaccine distribution and tally sheets	-	Yes	Yes	Yes	Yes	Yes	-	Yes	Yes	Yes
Unopened OPV kept at adequate temperature (0-8 °C)	Yes	Yes	Yes	Yes	Yes	Yes	Yes	Yes	Yes	Yes
Availability of enough frozen ice packs/ice for current session	Yes	Yes	Yes	Yes	Yes	Yes	Yes	Yes	Yes	Yes
Adequate knowledge of health workers regarding VVMs and unused OPV	Yes	Yes	Yes	Yes	Yes	Yes	-	Yes	Yes	Yes
Inquiry about vaccination status of all children<2 years from child's caretaker	Yes	Yes	-	-	-	Yes	-	Yes	-	Yes
Inquiry about acute flaccid paralysis in children<15 years from child's caretaker	-	Yes	-	-	-	Yes	-	-	-	-
Reminding child's caretaker for next round of NIDs	-	-	-	-	-	-	-	-	-	-
Adequate knowledge of child's caretaker about end results of polio and purpose of NIDs	-	-	-	-	-	-	-	-	Yes	-
**Mobile Teams**										
Correct storage of OPV (keeping vials dry and cold chain maintained as indicated by VVMs)	Yes	Yes	Yes	Yes	Yes	Yes	Yes	Yes	Yes	-
Correct administration of OPV (2 drops/child)	Yes	Yes	Yes	Yes	Yes	Yes	-	Yes	Yes	-
Covering of missed children on same day	Yes	Yes	Yes	Yes	Yes	Yes	Yes	Yes	Yes	Yes
Presence of at least one female member	Yes	Yes	Yes	Yes	Yes	Yes	-	Yes	-	Yes
Checking by supervisory staff	-	Yes	-	Yes	Yes	Yes	-	-	Yes	Yes
